# Systematically missing confounders in individual participant data meta-analysis of observational cohort studies

**DOI:** 10.1002/sim.3540

**Published:** 2009-02-16

**Authors:** The Fibrinogen Studies Collaboration

**Keywords:** meta-analysis, survival analysis, confounders, observational studies, missing covariates

## Abstract

One difficulty in performing meta-analyses of observational cohort studies is that the availability of confounders may vary between cohorts, so that some cohorts provide fully adjusted analyses while others only provide partially adjusted analyses. Commonly, analyses of the association between an exposure and disease either are restricted to cohorts with full confounder information, or use all cohorts but do not fully adjust for confounding. We propose using a bivariate random-effects meta-analysis model to use information from all available cohorts while still adjusting for all the potential confounders. Our method uses both the fully adjusted and the partially adjusted estimated effects in the cohorts with full confounder information, together with an estimate of their within-cohort correlation. The method is applied to estimate the association between fibrinogen level and coronary heart disease incidence using data from 154 012 participants in 31 cohorts.† Copyright © 2009 John Wiley & Sons, Ltd.

## 1. INTRODUCTION

Results from observational studies, such as epidemiological cohort studies, are susceptible to the distorting influence of confounders. These variables, through their association with the outcome of interest, can result in misleading inferences for the effect of other covariates unless they are properly adjusted for. Although including potential but unimportant confounders results in a loss of precision, inappropriately excluding them can lead to erroneous conclusions, and hence it is preferable to err on the side of caution by including all available possible confounders when modelling data [1].

Meta-analysis of observational studies using published data is fraught with difficulties: for example, different studies may define key variables differently, handle quantitative exposures differently (for example, with or without categorization), adjust for different confounders and use different analysis models [2]. Meta-analysis of individual participant data (IPD) avoids many of these problems [3]. However, one problem typically remains in IPD meta-analysis of observational studies: it is highly unlikely that all studies will provide information on the same set of potential confounders.

In such situations, two simple approaches are obvious: either use only those studies that provide full details of a set of potential confounders, and provide a statistical analysis that is fully adjusted for all potential confounders, or use all the available studies but omit some potentially important confounders. Although both are easily implemented, the first procedure has the limitation that it discards information and results in an inevitable loss of precision, while the second may omit important confounders and therefore be misleading. A compromise may therefore also be considered by making a judgement concerning which potential confounders to include while not disregarding too many studies.

In this paper we aim to improve on these simple methods by exploiting the relationship between fully and partially adjusted analyses and use all the available cohorts to make inferences about the fully adjusted effect. Studies that provide full details of all potential confounders can be used to obtain both fully *and* partially adjusted estimates, and hence can be used to ascertain the nature of the association between the two, while those that provide only a subset of confounders can be used to provide partially adjusted estimates alone. We propose a joint model for the fully and partially adjusted estimates. We allow for statistical heterogeneity between studies by using the standard bivariate random-effects model for meta-analysis [4–6] for the partially and fully adjusted estimates of effect. This enables inferences concerning both partially and fully adjusted effects to ‘borrow strength’ [6] from the other type of estimate. Most importantly, the fully adjusted estimate borrows strength from the studies that only provide partially adjusted estimates. The bivariate model also facilitates making inferences concerning the two types of effect simultaneously. A related idea is the ‘adaptation method’ suggested by Steyerberg and colleagues [7, 8] and this is discussed further in Section 8.

A two-stage approach will be adopted. At the first stage, partially and (where possible) fully adjusted estimates are obtained from each study, together with their standard errors; a key issue is estimating the ‘within-study’ correlation of the two estimates. At the second stage, the results are combined in a bivariate meta-analysis.

A two-stage approach is unavoidable for the meta-analysis of published time-to-event data [9, 10]. It has the disadvantage of implicitly making a quadratic approximation to the within-study log-likelihoods (i.e. assuming the within-study estimated effects are normally distributed), an approximation that is likely to be poor when studies have few events. With IPD, a one-stage approach to analysis is possible: in this a single model for all studies, typically incorporating study-level random effects, is fitted directly, thus avoiding the within-study quadratic approximation. One-stage approaches for IPD random-effects meta-analyses have been suggested for continuous [11], binary [12], ordinal [13] and time-to-event outcomes [14]. In this paper, we do not adopt a one-stage approach because of its computational complexity with time-to-event outcomes [14], because of the large size of our motivating data set, and because it is not clear how to encompass both fully adjusted and partially adjusted models. The two-stage approach retains the other advantages of IPD meta-analyses over their aggregate data counterparts, such as ensuring that all studies have the same variable definitions and the same analysis models, and enabling subgroup analyses [11].

The paper is set out as follows. In Section 2 we describe our motivating example, an IPD meta-analysis of 31 cohort studies relating plasma fibrinogen levels to time to coronary heart disease events [15]. Here only 14 cohorts provide information on all confounders, so 17 cohorts cannot provide fully adjusted estimates. In Section 3 our proposed model is described. In Section 4 procedures for estimating the within-study correlations are derived and in Section 5 the numerical implementation is discussed. Some illustrative analyses are performed in Section 6 and in Section 7 we return to the original data analysis and perform analyses more directly applicable to this. Section 8 summarizes our conclusions.

## 2. THE FIBRINOGEN DATA

We re-examine the database of our large collaborative IPD meta-analysis which explored the association between plasma fibrinogen and coronary heart disease in 31 cohort studies with 154 012 participants [15]. This was assessed using a proportional hazards (Cox) model, stratified by cohort, sex and (for the two cohorts that were randomized controlled trials) trial arm.

All 31 cohorts record whether or not coronary heart disease events occurred, and the times to event or censoring. They also provide details of every participant's fibrinogen level, age, smoking status, total cholesterol, systolic blood pressure and body mass index. Particular interest lies in the effect of participants' fibrinogen levels on coronary heart disease-free survival times, and the other covariates included in the models below represent potential confounders.

Only 14 cohorts give near complete data on participants' HDL cholesterol, LDL cholesterol, alcohol consumption, triglycerides and history of diabetes. A summary of the completeness of these additional covariates is provided in Table I. This table shows that cohorts generally have relatively few missing observations on variables that their designs intended to collect; the overwhelming majority of the missing observations are therefore systematically missing. There is however a single cohort that attempts to provide details of all five of the additional confounders but has much lower response rates for the cholesterol variables and triglycerides. In order to ensure consistency with our previous analysis [15], this particular cohort is treated as not providing details of these. A further issue is that total, HDL and LDL cholesterol levels are likely to be fairly collinear so only HDL and LDL cholesterol covariates, and not total cholesterol, were previously included in the full model [15]. Hence the covariates used in the partial models, using just the first set of covariates described above, were not quite a subset of those used in the full model.

**Table I:** Details of the completeness of the partially reported confounders.

Confounder	Number of cohorts	Number of participants in these cohorts	Per cent reported in these cohorts
HDL cholesterol	23	109789	99.3
LDL cholesterol	20	98263	98.0
Alcohol consumption status	25	120909	98.1
Triglycerides	18	91226	99.9
History of diabetes	28	123257	97.0
All of the above	14	75 899	94.8

We [15] previously performed two series of analyses: the first using information from all 31 cohorts, but adjusting only for covariates in the first set, and the second adjusting for covariates in both sets, but using just the information from the 14 cohorts that adequately record the necessary details. The intention here is to produce an analysis that takes into account all of the various potential confounders but also uses information from all 31 cohorts.

## 3. A BIVARIATE MODEL FOR MISSING CONFOUNDERS

In this section, a model is developed for the scenario where all cohorts provide the same subset of confounders and only some cohorts provide all of the confounders. It is also assumed that the response is the time until event, and that the proportional hazards model is appropriate. For the purposes of model development, we assume that the full model uses all the available covariates, and return later in Section 7 to the issue concerning the omission of total cholesterol from this model. We further assume no nonsystematically missing values within cohorts and also address this issue in Section 7.

### 3.1. Modelling individual participants within cohorts

In a particular cohort, let *X_S_* denote the vector of an individual participants' stratifying covariates (sex and trial arm in our data), let *X*_1_ denote the column vector of other covariates that are also observed by all cohorts (including the covariate of particular interest) and let *X*_2_ denote the column vector of covariates that are only observed by some cohorts.

For each cohort where *X*_2_ is observed we assume the full proportional hazards model for the time to event,


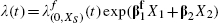
(1)

and we similarly assume for the partial model, i.e. without the covariates *X*_2_, that


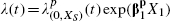
(2)

where λ denotes the hazard function. This notation emphasizes the difference in the parameters **β_1_** and the baseline hazard functions in the two models: in the full model these are denoted with a superscript *f* indicating quantities that are *fully* adjusted for (i.e. take into account all the covariates *X*_1_ and *X*_2_) while in the partial model the superscript *p* denotes quantities that are only *partially* adjusted, as they do not take into account the covariates *X*_2_. Both models apply to a particular cohort, and it is anticipated that cohorts will have different baseline hazards. Although both the full and partial models cannot simultaneously be true, unless **β_2_ = 0**, both are likely to provide adequate descriptions of the data [16]. Note that bold font is used for row vectors of parameters in these models to distinguish between these and their first entries in the notation that follows.

We can obtain estimates 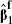, 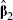 (from the full model 1) and 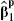 (from the partial model 2) for each cohort that provides details of *X*_2_, by maximizing the partial likelihood in the usual way [17]. For those cohorts that do not provide details of *X*_2_, we can only obtain the corresponding estimate from model (2).

Let the first entry in *X*_1_ denote the covariate of particular interest. We are therefore interested only in inference regarding the first parameter in the vectors 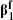 and 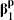; the others represent potential confounders. The corresponding estimates are the first entries in 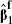 and 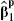 which will be denoted by 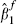 and 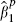.

### 3.2. Between-cohorts model

We assume *for any given cohort* that


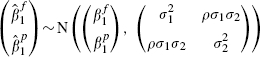
(3)

where we assume that 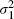, 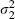 and ρ are fixed and known, a conventional assumption when using bivariate models in meta-analysis [5, 6] and a generalization of assuming that the within-cohort variances are fixed and known in more usual univariate analyses [18, 19]. In practice, however, these values must be estimated by standard methods: the variances are provided by the output of proportional hazards regression in standard statistical packages, and obtained from the observed information matrix [20, p. 41]. The difficulty lies in estimating ρ, and some approaches for obtaining this are suggested in Section 4.

The underlying 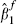 and 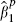 may vary from cohort to cohort. We assume that this variation can be modelled as


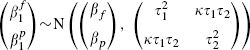
(4)

providing the marginal bivariate normal distribution for the cohort in question as


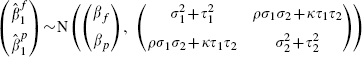
(5)

where we anticipate, but do not require, that both ρ and κ will be positive. Equation (5) is simply the standard bivariate model for meta-analysis, where the two outcomes are the partially and fully adjusted effects. This is an innovative use of this standard model, as more usually the outcomes are not defined so similarly (typically they are notably different types of patient responses). Despite this, the central limit theorem implies that model (3) provides a good approximation for large cohorts such as these and, combining this with model (4), which describes the between-cohort variation, the bivariate random-effects model is a natural choice for data such as these.

In a standard univariate meta-analysis of the fully adjusted effect of fibrinogen level, using just the cohorts that provide the necessary information on all confounders, the marginal model for 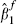 from (5) is assumed and information from 17 cohorts is simply discarded. This comment applies to all the analyses performed using the bivariate model below.

### 3.3. The log-likelihood function of the fibrinogen data

For cohorts that do not provide *X*_2_, only the partial model (2) is fitted, 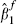 is unobserved and, assuming that this is missing at random (MAR), the marginal distribution of 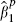 from model (5) alone is required.

The resulting log-likelihood function of the data, i.e. the fully and partially adjusted estimates from the 14 cohorts that give full confounder information, and the partially adjusted estimates from the remaining 17 cohorts, obtained as described in Section 3.1, is


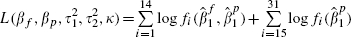
(6)

where the bivariate and marginal densities, *f_i_*(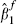, 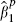) and *f_i_*(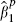), are obtained directly from distributions (5), the first and second summations in (6) being over the cohorts that provide *X*_2_, and those that do not, respectively. This likelihood involves five parameters, but β_*f*_ is of primary interest. Although the cohorts that fail to report *X*_2_ do not provide direct evidence relating to β_*f*_, they provide indirect information via their partially adjusted estimates and their assumed association with the fully adjusted estimates. The bivariate random-effects model therefore allows inferences concerning the fully adjusted effect to borrow strength from cohorts where fully adjusted estimates are unavailable, as explained in the introduction. The bivariate model also enables us to examine the nature of the relationship between the two types of effects.

Missing estimates are not imputed by this procedure, but the relationship between the fully and partially adjusted estimates, for the cohorts where both estimates are available, is assumed to apply to those where only partially adjusted estimates can be obtained. Since a bivariate normal model is adopted this association is assumed to be linear, so that the method bears some similarities to the approach of Riley *et al.* [21], who impute missing estimates and standard errors from linear trends in the context of a sensitivity analysis.

Inferences for the partially adjusted β_*p*_ are also made when fitting the bivariate model, which makes use of the fully adjusted estimates, although this borrowing of strength is likely to be very limited, as all 31 partially adjusted estimates are available. Once the fully and partially adjusted estimates have been obtained, the methodology therefore becomes a fairly standard application of the bivariate random-effects model for meta-analysis, but with one very particular difficulty: the within-cohort correlations are assumed known but need to be estimated. Some novel approaches are therefore developed for this purpose in the next section.

## 4. ESTIMATING THE WITHIN-COHORT CORRELATION ρ

Although values of ρ are estimated for each cohort, once evaluated these are regarded as fixed and known. We therefore suppress the emphasis that ρ is an estimate in the notation that follows.

### 4.1. A nonparametric bootstrap estimate, ρ_*b*_

Nonparametric bootstrapping [22] is probably the simplest, but slowest, procedure for obtaining an estimate of ρ. For each cohort that provides details of *X*_2_, participants can be sampled with replacement providing a bootstrap sample, where for each sampled individual we record all their various details: their time to event, all covariates and note whether or not they were censored. For each bootstrap sample, the ordered pair of estimates 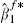 and 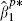 provide the required bootstrap replication. The estimate ρ_*b*_ is then obtained as their sample correlation.

### 4.2. An analytical estimate, ρ_*a*_

An approximate analytical estimate of ρ is also possible. This procedure is akin to the approach suggested by Steyerberg *et al*. [7, 8], as an algebraic connection between the fully and partially adjusted estimates is utilized. We first consider the linear regression case, in which an exact expression is possible, and then extend this to the proportional hazards model. Consider the analogous linear regressions 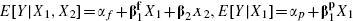 and *E* [*X*_2_|*X*_1_ = α + γ*X*_1_, where the various α parameters represent model intercepts; note that the regression of *X*_2_ on *X*_1_ is a multiple multivariate regression and therefore that α and γ denote a vector and a matrix, respectively. Evaluating *E* [*Y*|*X*_1_] = *E*_*X*_2_|*X*_1__ [*E* [*Y*|*X*_1_, *X*_2_]], and equating terms in *X*_1_, provides 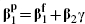.

The corresponding identity also applies to maximum likelihood estimates, a result that may be proved by examining the normal equations resulting from the various linear regressions. In particular, using 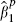 and 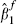 to denote the first entries in the corresponding row vectors, 
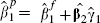
 where 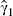 denotes the first column of 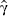. This vector contains the estimated regression coefficients of *X*_2_ on the first term in *X*_1_ i.e. the covariate of particular interest. This means that 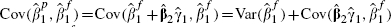.

Since 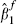 and 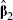 are estimated coefficients from the regression of *Y* on *X*_1_ and *X*_2_, their properties depend on the distribution of *Y* conditional on both *X*_1_ and *X*_2_; similarly 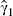 is a vector of estimated coefficients whose properties depend on the distribution of *X*_2_ conditional on *X*_1_. The random variables 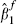 and 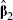 are therefore functions of the random variable (*Y*|*X*_1_, *X*_2_) and 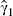 is a function of (*X*_2_|*X*_1_). By definition, (*Y*|*X*_1_, *X*_2_) and (*X*_2_|*X*_1_) are independent and hence 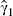 is independent of both 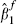 and 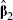. Hence, 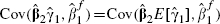. Thus using the approximation 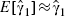, the covariance of the fully and partially adjusted estimates can also be obtained approximately, and is evaluated as


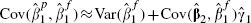
(7)

Since all the entries in the 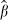 vectors in the right-hand side of (7) are from the full model, i.e. the model including *X*_2_ as a covariate, their variances and covariances may be obtained when fitting this model using any standard method.

The above applies to a linear regression, but in our application we assume the proportional hazards model for survival time *T* where, in addition to *X*_1_ and *X*_2_, we have stratifying variables *X_S_*. We assume a linear regression for *X*_2_, *E*[*X*_2_|*X*_1_, *X_S_*] = α + γ*X*_1_ + δ*X_S_*. From equation (1), we have


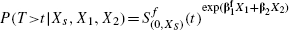


where 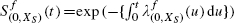 denotes the baseline survivor function for the full model, stratified by *X_S_* as before. Interpreting *P*(*T*>*t*) as the expectation of the event *T*>*t*, we now use the iterated expectation formula *E*[*A*|*B*] = *E*[*E*[*A*|*B, C*]|*B*], with *A* = the event *T*>*t*, *B* = (*X_s_*, *X*_1_) and *C* = *X*_2_, to show that


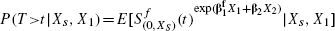


Next we use the simple Taylor Series expansion *E*[*g*(*V*)|*W*] ≈ *g* (*E*[*v*|*W*]), with


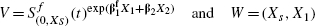


to show


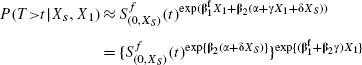


which is of the form of the partial model (2) with 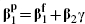, and where other terms have been absorbed into the baseline hazard function.

We therefore suggest that 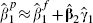 be used as an approximation and hence that (7) be used to obtain the within-cohort covariance as for linear regression. An estimate of 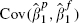 can be therefore be obtained and the within-cohort correlation ρ_*a*_ can be obtained as 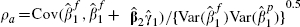.

### 4.3. Modified analytic correlations, ρ_*m*_

A potential difficulty is that the analytic approach in Section 4.2 provides no assurance that the correlations lie between −1 and 1; as shown below, three fibrinogen cohorts provide analytic correlations that are slightly greater than one.

A modification of the analytical approach that avoids such estimates can be developed by defining 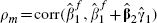. This requires the calculation of 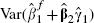. The simplest way to evaluate this variance is to derive the covariance matrix of the six terms that comprise the summation 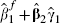 and evaluate 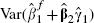 directly from this. Noting that 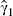 is independent of 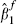 and 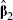, the entries of this covariance matrix can be evaluated using the identity Cov(*A*_1_*B*_1_, *A*_2_*B*_2_) = Cov(*A*_1_, *A*_2_)Cov(*B*_1_, *B*_2_) + *E*(*A*_1_)*E*(*A*_2_)Cov(*B*_1_, *B*_2_) + *E*(*B*_1_)*E*(*B*_2_)Cov(*A*_1_, *A*_2_), assuming that **A** and **B** are independent; expected values are approximated by point estimates and the necessary covariances are estimated when fitting the full proportional hazards and the multiple linear regression models.

Note that we continue to use the direct estimate of 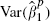 not 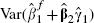 for the variance 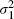 in order to follow the convention that within-cohort variances are obtained using standard methods.

### 4.4. Comparison of the procedures for estimating ρ

The procedures for estimating ρ provide contrasting approaches. In particular, the bootstrap is computationally expensive, requiring considerable resampling and the repeated fitting of models involving large numbers of participants. Estimates of ρ for the 14 fibrinogen cohorts can however be obtained in minutes, rather than hours, using several hundred bootstrap replications. A technicality here is that the resampling almost inevitably results in ties; Efron's method [23] was used for handling these, although other standard methods also provide very similar estimates of ρ for the fibrinogen data.

The limitation of the analytical approaches is that they involve an algebraic approximation, and it is difficult to ascertain how accurate this is. The analytical approaches should be used only when exactly the same participants are used to fit both full and partial models, as this is required so that 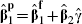 in the analogous linear regression, which motivates the approximation. For example, some participants in the 14 fibrinogen cohorts that provide details of *X*_2_ have some missing covariates in *X*_2_ but provide complete information for *X*_1_; including these participants when fitting partial models but then omitting them in full models invalidates the theory. A further issue raised by the analytic approaches is that it is required that the partial model involves a subset of covariates from the full model. These issues do not present problems for the bootstrap procedure.

To summarize, no single method is preferable to the others on all grounds, so they are compared in Section 6.

## 5. NUMERICAL IMPLEMENTATION

*R* software was used throughout. The ‘survival’ package was used to fit all the necessary proportional hazards models and the ‘sample’ command was used to sample random rows, with replacement, from the data frame for the bootstrap replications required when evaluating ρ_*b*_. Having estimated the within-cohort correlations, following van Houwelingen *et al*. [24], maximum likelihood estimation was performed and confidence intervals were obtained from the profile log-likelihood. It should however be noted that alternative estimation procedures, such as restricted maximum likelihood (REML), are also possible but are unlikely to make much difference here as the sample size is relatively large.

The necessary maximizations of the log-likelihood (6) were performed numerically using the ‘optim’ command, with the quasi-Newton ‘BFGS’ method, after transforming the variance and correlation parameters so that the transformed values lie along the whole real line. By specifying particular parameters as further arguments to be passed to the log-likelihood, these can be constrained to particular values and hence profile log-likelihoods can be also be obtained numerically. The command ‘fdHess’, from the ‘nlme’ package, provides a Hessian matrix that can be evaluated at the maximum likelihood estimates and then inverted in order to produce the observed information matrix from which standard errors can be obtained. When maximizing the resulting log-likelihoods in this manner, it was necessary to reduce the ‘ndeps’ vector, which denotes the step sizes for the finite-difference approximation to the gradient, from its default value of 10^−3^ to 10^−5^. Although the fibrinogen data are not freely available, illustrative *R* code for performing the analysis is available upon request from the first member of the writing committee.

## 6. SIMPLE ANALYSES OF THE FIBRINOGEN DATA

Three complications arise with this database. First, some individuals in ‘complete’ cohorts do not in fact have complete data on *X*_2_. In order to apply the analytic methods for obtaining correlations in Section 4, we exclude these individuals from the estimation of both full and partial models (‘complete-case analysis’) but in Section 7 we alternatively include them in the partial model. Second, many ‘partial’ cohorts have data on some variables in *X*_2_, as shown in Table I. We ignore this information but consider its use in the discussion. Third, the partial model previously fitted was not quite a submodel of the full model, as only the partial model includes total cholesterol as a covariate. In order to apply the methods of Section 4 we drop total cholesterol from the partial model in this section; in Section 7 we alternatively include total cholesterol in this model.

### 6.1. Cohort-specific estimates for the fibrinogen data

The estimates 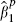 and 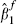 of the effect of fibrinogen level, their within-cohort standard errors, σ_1_ and σ_2_, and the correlations described above, for the 14 cohorts that provide the necessary information, are shown in Table II. The estimates and variances were obtained from standard proportional hazards model output and the correlations were obtained using the bootstrap (Section 4.1, with 500 bootstrap replications), the analytical (Section 4.2) and the modified analytical (Section 4.3) procedures. A 95 per cent confidence interval for a bootstrap within-cohort correlation of 0.95, based on Fisher's transformation, is (0.941, 0.958), indicating that 500 bootstrap replications are sufficient to accurately estimate the correlations.

**Table II:** The estimates 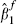 and 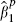 of the log hazards ratio of the effect of fibrinogen level, their within-cohort standard errors and correlations, for complete-case analyses of the 14 fibrinogen cohorts that provide the necessary details of *X*_2_.

Cohort	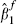	σ_1_	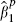	σ_1_	ρ_*b*_	ρ_*a*_	ρ_*m*_
1	−0.353	0.381	−0.188	0.387	0.861	0.984	0.970
2	0.334	0.088	0.425	0.085	0.971	0.981	0.961
3	0.309	0.132	0.394	0.129	0.963	0.978	0.962
4	0.324	0.198	0.435	0.191	0.963	0.988	0.976
5	0.400	0.296	0.543	0.272	0.961	0.999†	0.980
6	0.149	0.104	0.151	0.103	0.988	0.999†	0.994
7	0.262	0.120	0.327	0.117	0.974	0.996	0.982
8	0.436	0.310	0.541	0.312	0.945	0.957	0.974
9	0.337	0.113	0.451	0.108	0.965	0.998	0.976
10	0.474	0.143	0.609	0.137	0.952	0.999†	0.982
11	0.110	0.086	0.159	0.085	0.985	0.984	0.985
12	0.413	0.065	0.532	0.064	0.963	0.982	0.970
13	0.213	0.078	0.262	0.077	0.964	0.976	0.969
14	0.062	0.175	0.129	0.170	0.962	0.989	0.976

† Correlations marked were estimated to be more than unity, and hence have been truncated at 0.999.

All values of ρ in Table II are large and positive, reflecting the similar nature of the two types of estimates. However, it is interesting to note that values of ρ_*a*_ are generally greater than the corresponding values obtained by bootstrapping and, as noted above, three of these were estimated to be very slightly greater than one. These were truncated to 0.999. For scenarios where the estimates are not so highly correlated, this truncation is unlikely to be necessary suggesting that this analytical solution is likely to perform more satisfactorily in such cases.

**Figure 1:**
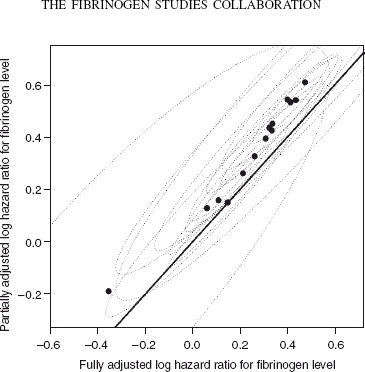
Fully and partially adjusted estimated effects of fibrinogen level and corresponding 95 per cent confidence intervals, using the bootstrap within-cohort correlations. The line of equality is also shown.

The pairs of estimates 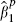 and 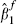 are also plotted in Figure 1, where the dotted lines indicate cohort specific 95 per cent confidence regions, obtained using model (3) and the bootstrap within-cohort correlations; the bootstrap correlations were used in this figure as they are generally smaller than the analytic ones, and provide more attractive and somewhat less squashed ellipses. The line of equality indicates that all the partially adjusted estimates are greater than the corresponding fully adjusted estimate.

The trend in Figure 1 appears linear, lending credence to the assumption of bivariate normality across cohorts. Almost all the confidence regions overlap indicating that the results are broadly comparable across cohorts. However, it should not be supposed from this plot that the random effects model in (4) is not required and that a fixed effects model is appropriate: the univariate χ^2^ heterogeneity statistics for the fully and partially adjusted estimates shown in Figure 1 are 17.8 (*I*^2^ = 0.27) and 28.3 (*I*^2^ = 0.55), on 13 degrees of freedom, respectively. Although the first of these χ^2^ statistics is not significant, the second of these provides a *p*-value of 0.008 when testing the null hypothesis that the partially adjusted estimates are homogenous.

The estimates 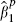 from the remaining 17 cohorts are shown in Table III. Cohort 31, although providing an apparently unusual estimated effect is also much the smallest cohort (only 418 participants), and there are no obvious signs of outliers. Removing this very small cohort makes virtually no difference to the resulting inferences, although very slightly larger effects of fibrinogen level are obtained if this is discarded.

**Table III:** The estimates 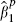 of the log hazards ratio of the effect of fibrinogen level and their within-cohort standard errors, for the 17 fibrinogen cohorts that do not provide full details of *X*_2_.

Cohort	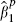	σ_2_
15	0.438	0.342
16	0.484	0.115
17	0.154	0.120
18	0.660	0.252
19	0.290	0.083
20	0.333	0.117
21	0.122	0.147
22	0.666	0.349
23	0.219	0.053
24	0.354	0.126
25	0.553	0.148
26	0.338	0.087
27	0.439	0.083
28	0.215	0.045
29	0.304	0.278
30	0.429	0.108
31	1.190	0.499

### 6.2. A complete-case meta-analysis using ρ_*a*_

In this section the analytic within-cohort correlations are used. The implications of using other within-cohort correlations are explored in Section 6.3.

**Figure 2:**
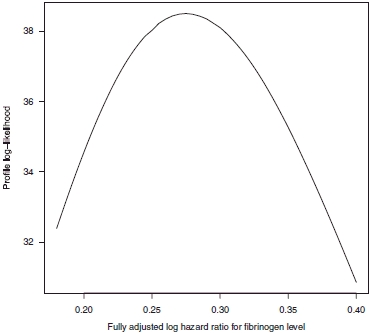
Profile log-likelihood plot for β_*f*_ using the analytic within-cohort correlations, shown in column 7 of Table I.

The profile log-likelihood was adopted to make inferences about β_*y*_. In computing this, we fix the value β_*y*_ and maximize the log-likelihood (6) over the remaining four parameters, subject to the constraints that τ_1_≥0, τ_2_≥0 and —1≤κ≤1. This profile log-likelihood is shown in Figure 2.

**Figure 3:**
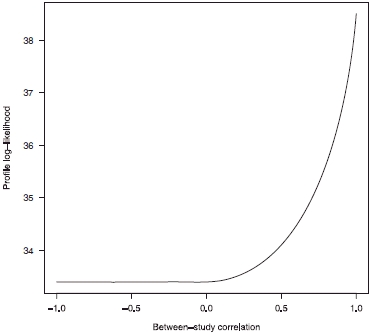
Profile log-likelihood plot for κ using the analytic within-cohort correlations, shown in column 7 of Table I.

The maximum likelihood estimate 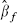 is 0.275 and a corresponding 95 per cent confidence interval, given by values of 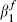 that provide a profile log-likelihood within 1.96^2^/2 of its maximum, is (0.223, 0.332). Figure 2 masks an important finding that 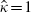 and hence this parameter estimate is located at the edge of the parameter space; indeed this is the case for *all* the analyses described below. Furthermore the values of κ required to maximize the log-likelihood for all the values of β_*f*_ used to draw Figure 2 are greater than 0.9999. The corresponding profile log-likelihood plot in terms of κ is shown in Figure 3, which shows that there is strong evidence for a large and positive between-cohort correlation.

This finding presents difficulties when using information matrices to obtain confidence intervals, although by constraining κ = 1 we may easily obtain confidence intervals in this way. This comment applies for all the analyses below. This finding is not particularly surprising, given the analogous nature of fully and partially adjusted estimates and the finding of Riley *et al*. [6] that estimates of between-cohort correlation frequently lie at the edge of the parameter space. In particular, there is not a very large number of cohorts providing information concerning this parameter, and reasonably large within-cohort variances, relative to the between-cohort variation, which are features of data that tend to give rise to 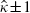 [6].

### 6.3. Comparison of results

The results for the different estimates of ρ are shown in Table IV, where results for both the fully and partially adjusted effect of fibrinogen level are shown and ‘modified’ refers to the modified analytic within-cohort correlations described in Section 4.3. Estimates of K are not tabulated here because, as noted above, 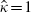 for all models fitted.

**Table IV:** Parameter estimates for simple analyses where partial models omit total cholesterol and include only complete cases.

Correlations	β_*f*_	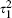	β_*p*_	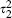
Bootstrap	0.271 (0.026)	0.005 (0.004)	0.346 (0.030)	0.011 (0.006)
Analytic	0.275 (0.027)	0.006 (0.004)	0.358 (0.031)	0.013 (0.006)
Modified	0.272 (0.027)	0.005 (0.004)	0.350 (0.030)	0.011 (0.006)

‘Correlations’ refers to the method used to obtain within-cohort correlations. Standard errors, obtained from the observed information matrix, having constrained κ = 1, are shown in parentheses.

Constraining κ = 1 and obtaining confidence intervals using this reduced model, and the observed information matrix, provides very similar inferences for the effect of fibrinogen level as when using the profile log-likelihood and avoids the difficulty that 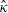 lies at the edge of the parameter space. The standard errors of all estimates in Table IV are shown in parentheses, using the observed information matrix and this reduced model. It should be noted that the extreme estimate of K tends to lead to inflated estimates of between-cohort variance [6] but these are not insubstantial and all the various models in Table IV provide similar results. It is interesting to note that estimates of between-cohort variance of the partially adjusted effects are much greater than the corresponding estimates for the fully adjusted effects, suggesting that the additional confounders incorporated into the full model explain some of the heterogeneity in the estimates of the effect of fibrinogen level.

We conclude that the choice of method for estimating ρ is not important in these data. Using the bootstrap within-cohort correlations provides a 95 per cent confidence interval of (1.24, 1.38) for the fully adjusted hazard ratio for fibrinogen level. The average fibrinogen level in the sample is 3.02 and the upper and lower quartiles are 2.47 and 3.47, respectively, indicating that participants with fibrinogen levels in the top quartile are at considerably more risk of a coronary heart disease event than those in the lower quartile.

### 6.4. Comparison with analyses of ‘full’ cohorts

The bivariate model gives estimated fully adjusted effects of fibrinogen in the range 0.259–0.275, with standard errors of around 0.027. For comparison, a simple univariate random-effects meta-analysis, using just the 14 cohorts that provide the necessary information (and hence using just the data in columns 2 and 3 of Table II), provides a point estimate of 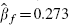 with a standard error of 0.038. Furthermore, a standard bivariate random-effects meta-analysis using just these 14 cohorts and the bootstrap within-cohort correlations (i.e. using only the data in columns 2–6 of Table II) gives 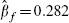 with a standard error, obtained in the same way as in Table IV, of 0.041. These much larger standard errors indicate that the extra information incorporated into the model developed here has been worthwhile in estimating the effect of fibrinogen, as the reduction in the standard error using the proposed procedure is around 30 per cent. It is also interesting to note that the standard error resulting from using the bivariate random-effects model for just the 14 cohorts that provide details of *X*_2_ is very similar to that from the usual univariate meta-analysis of the fully adjusted effects. This indicates that little or no ‘borrowing of strength’ from unadjusted estimates of effect occurs for this example unless partially adjusted estimates from the remaining 17 cohorts are also used in the analysis. This is not surprising, as a number of previous articles highlight that, given complete data, there is little benefit of bivariate over univariate meta-analysis [6, 25, 26].

## 7. EXTENDED ANALYSES

The analyses in the previous section are not entirely in the spirit of those performed previously [15]. First, some individuals in ‘full’ cohorts had incomplete *X*_2_. We excluded these individuals from estimation of both full and partial models in Section 6, which we called a complete-case analysis. Now we will follow the previous analysis by including these individuals in estimating the partial model.

Second, we have omitted total cholesterol from the partial model. Now we follow the previous analysis by including it.

These changes invalidate the assumptions underlying the analytic approaches for estimating ρ. We therefore use bootstrap within-cohort correlations in this section. Here the bootstrap replications simply include total cholesterol and incomplete cases when fitting partial models but are otherwise obtained as before. Making the above changes slightly modifies the cohort-specific values of 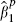 and the accompanying standard errors, but leaves 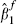 unchanged. Following the same procedure as before provides 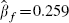 and a 95 per cent confidence interval from the profile log-likelihood is (0.208, 0.314). This is broadly similar to the analyses above but a slightly smaller effect of fibrinogen is inferred; very similar inferences are also made by assuming that κ = 1 and using the observed information matrix, as shown by the second set of results in Table V.

**Table V:** Parameter estimates for extended are obtained by analyses. All within-cohort correlations are obtained by bootstrapping.

Total cholesterol	Complete-case	β_*f*_	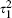	β_*p*_	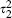
Yes	Yes	0.263 (0.026)	0.005 (0.003)	0.319 (0.028)	0.008 (0.005)
Yes	No	0.259 (0.026)	0.005 (0.004)	0.320 (0.026)	0.006 (0.004)
No	No	0.269 (0.027)	0.005 (0.004)	0.341 (0.027)	0.008 (0.004)

Affirmative ‘Complete-case’ and ‘Total cholesterol’ indicate that a complete-case analysis has been performed, and that total cholesterol is included in partial models, respectively. Standard errors, obtained from the observed information matrix, having constrained κ = 1, are shown in parentheses.

### 7.1. Comparison of results

The choices concerning whether or not to include incomplete cases and total cholesterol when fitting partial models are somewhat arbitrary and the implications of these decisions are now explored. The results for models that exclude both total cholesterol and incomplete cases have been summarized in Table IV; the corresponding results for the other three possibilities, using bootstrapping to obtain the within-cohort correlations, are summarized in Table V. A comparison of Tables IV and V indicates that the inferences are not particularly sensitive to these choices when fitting partial models.

### 7.2. A simplified model

**Figure 4:**
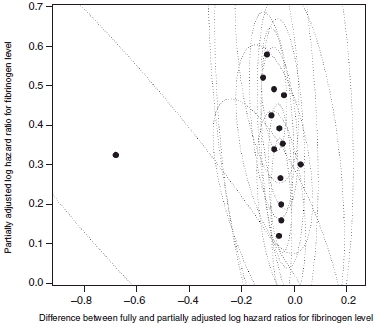
The difference between fully and partially adjusted, and partially adjusted, estimated effects of fibrinogen level and corresponding 95 per cent confidence intervals. Note that the partially adjusted estimates shown here adjust for total cholesterol, and hence are not quite the same as those shown in Figure 1 or Table I.

A plot similar to Figure 1, *but with the difference between fully and partially adjusted estimates on the horizonal axis, and where partial models include total cholesterol*, is shown in Figure 4. The differences between the fully and partially adjusted estimates (horizontal axis of Figure 4) appear homogenous, and the usual χ^2^ heterogeneity statistic of these differences is just 8.0 on 13 degrees of freedom. This apparent homogeneity is however highly sensitive to the estimated within-cohort correlations, so although not too much emphasis should be placed on this finding, this does suggest a simpler model that adequately describes the data. The point estimate 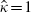 is obtained for all the various models, as noted above, and assuming that both κ = 1 and 
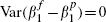
 is equivalent to assuming that both κ = 1 and 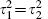 in model (5). Hence a much simpler model that appears to describe the data well is model (5) subject to these two constraints.

Using the data shown in Figure 4, this model provides a point estimate 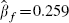 with a standard error of 0.027. This is very similar inference to the analyses performed above, so little is gained by this simplification: the change in deviance of this model, compared with the full model where κ, 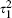 and 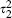 are allowed to take any value in their joint parameter space, is just 1.1 on 2 degrees of freedom.

## 8. CONCLUSIONS

Observational studies are likely to differ in terms of the information that they provide and are particularly susceptible to the influence of confounders. Provided that fully and partially adjusted estimates are kept distinct however, and assuming that at least some studies provide enough information to produce fully adjusted estimates, the methodology we have developed can be used or modified to incorporate data from all available cohorts. Similar methods are likely to be useful in other individual participant data (IPD) meta-analyses such as the million-person Emerging Risk Factors Collaboration [27].

Various extensions of the model are possible. For example, this kind of procedure could be used for other types of outcome, such as continuous and binary variables, or for other types of study, such as case–control studies. Furthermore, the fibrinogen data were analysed as providing two types of cohorts: those that provide adequate details of *all* the extra variables and those that do not. In other scenarios there may be much more complicated patterns of missingness and Table I shows that this distinction between the two types of fibrinogen cohort is a simplification. In other examples, further dimensions may be needed than the bivariate model suggested here allows. For example, one could fit models containing *X*_1_, *X*_1_ and *X*_2_, *X*_1_ and *X*_3_ and *X*_1_, *X*_2_ and *X*_3_, and perform a four-dimensional meta-analysis. Whether the additional model complexity is justified by increases in precision is a topic for further research.

An alternative way to estimate the within-cohort correlation is to estimate the unadjusted and adjusted Cox regressions simultaneously. Individuals from cohorts with complete data each contribute two records to this analysis, whereas individuals from other cohorts contribute only one record. The within-cohort correlation is obtained from the robust variance–covariance matrix of Wei *et al*. [28], which allows for dependence of different records on the same individual. This method provided similar correlations and inferences to those in Tables II, IV and V (results not shown). For situations where they are computationally feasible, the bootstrap correlations are perhaps preferable in practice because no linear approximation is required to derive them. Even if within-cohort correlations provided by alternative methods differed more notably, a recent simulation study concludes that a variety of types of errors when approximating within-cohort correlations have little impact on the estimation of the population means if there is complete data [25]. Here we borrow strength from 17 cohorts with incomplete data, however, and the correlation between the partially and fully adjusted estimates is crucial to the procedure adopted.

A recent proposal [29] for fitting a bivariate normal meta-analysis model, without estimating the within-cohort correlations, was not adopted as this does not separately reflect the within and between-cohort correlations, on which the borrowing of strength so critically relies when analysing the fibrinogen data in this way. The within-cohort correlations can be obtained with effort and ‘If practitioners are fortunate to have the within-study correlations available, or if they can be assumed zero, then we recommend that they still perform a bivariate random-effects meta-analysis using the general model’ [29] (model (5), as used here). Despite this, borrowing of strength can mostly be achieved without separating the within and between-cohort correlations and a referee pointed out that fitting this alternative model resulted in a similar estimate and confidence interval for the fully adjusted effect of fibrinogen. Those who wish to avoid estimating the within-cohort correlations altogether therefore have a viable alternative to consider.

Simply assuming that all the within-cohort correlations ρ = 1 provides very similar inferences for the fibrinogen data, and one might consider assuming ρ = 1 in order to quickly obtain some indicative results. Reparameterizations of the model, in order to avoid estimates 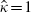, were also considered. In particular, a bivariate normal model for 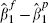 and 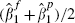 was examined but this provided 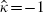 for almost all the types of analyses described above, and hence did not avoid the difficulties associated with estimating this correlation. A further extension is to consider the possibility of addressing measurement error when measuring fibrinogen levels [30].

Our method assumes that confounders unmeasured in a particular cohort are MAR: that is, there is no systematic difference in 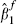 between studies that do and do not measure *X*_2_, once we allow for differences in 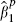. It is possible, but not likely, that some studies did not measure *X*_2_ because they thought it was not an important confounder in their study (which might contradict MAR).

By contrast, the standard univariate method of analysing the cohorts that measured *X*_2_ assumes that the confounders are missing completely at random: that is, there is no systematic difference in 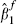 between cohorts that do and do not measure *X*_2_. This seems much less plausible, since for example the studies that did not measure *X*_2_ may be earlier studies that used alternative methods including different ways to measure the exposure of interest.

Combining unadjusted and adjusted estimates was previously proposed by Steyerberg and colleagues [7, 8]. They started with just three estimates: unadjusted and fully adjusted estimates from IPD, and an unadjusted estimate from published literature. Their proposed estimate of the fully adjusted coefficient equals the fully adjusted estimate from the IPD plus an ‘adaptation factor’ times the difference between the unadjusted estimates. The adaptation factor is computed from the observed standard errors and within-study correlations to minimize the variance of the resulting ‘adapted estimate’. One could extend this approach here, obtaining fully adjusted ‘individual participant’ estimates by conducting a bivariate meta-analysis using the 14 cohorts that provide full confounder information, and replacing the unadjusted estimate from published literature with the result of a univariate meta-analysis of the remaining 17 cohorts. If fixed-effect meta-analyses are performed then the adapted estimate turns out to be the same as a fixed-effect version of our procedure (i.e. setting τ_1_ = τ_2_ = 0 in equation (4)). This equivalence is lost if random-effects models are used. Our method has the advantages of greater transparency, because the model is clearly stated, and greater statistical efficiency because 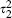 is assumed equal across the two subsets of cohorts. Our procedure also has the benefit of ease of computation, because only one bivariate random-effects meta-analysis is needed, and facilitates an entirely likelihood-based approach.

The methodology developed here could also be applied if some cohorts provide IPD while others provide only aggregate results, for example just one of the estimated effects and accompanying standard error. This is the scenario specifically considered by Steyerberg *et al*. [7, 8] and Riley *et al*. [31] provide a recent review of such methods. If necessary, one might assume that aggregate within-cohort correlations are comparable to those where the IPD are available. The assumption that estimated effects are MAR becomes a much stronger assumption when using aggregate data in this way however, as the publication of a particular analysis might depend on the results obtained, rather than just the availability of the covariates.
